# Vive la résistance!

**DOI:** 10.7554/eLife.02387

**Published:** 2014-03-04

**Authors:** Steven J Sandler

**Affiliations:** 1 **Steven J Sandler** is in the Department of Microbiology, University of Massachusetts, Amherst, United Statessandler@microbio.umass.edu

**Keywords:** DNA repair, ionizing radiation, evolution, extremophile, mutation, *E. coli*

## Abstract

In vitro evolution experiments reveal that single mutations in three genes can increase the ability of *E. coli* to survive ionizing radiation by a factor of 1000.

**Related research article** Byrne RT, Klingele AJ, Cabot EL, Schackwitz WS, Martin JA, Martin J, Wang Z, Wood EA, Pennacchio C, Pennacchio LA, Perna NT, Battista JR, Cox MM. 2014. Evolution of extreme resistance to ionizing radiation via genetic adaptation of DNA repair. *eLife* **3**:e01322. doi: 10.7554/eLife.01322

**Image** *E. coli* can evolve to become more resistant to ionizing radiation

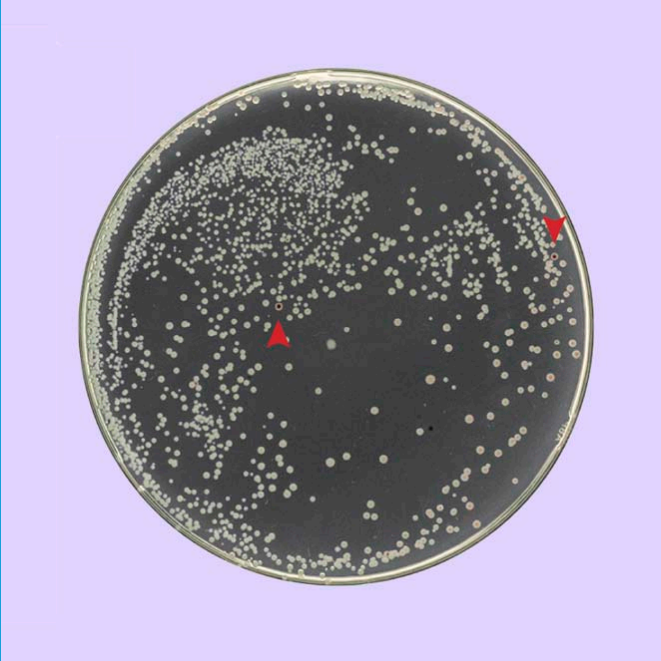

We all have DNA, and we need to replicate this DNA in order to grow and reproduce. Critically, however, we also need to protect our DNA from damage, or to repair it when it gets damaged. Sources of damage include certain chemicals produced during metabolism and various forms of radiation. Some DNA repair pathways are relatively simple: an enzyme called photolyase, for example, can repair the damage caused by exposure to ultraviolet radiation from the sun in a single reaction. However, it takes many different proteins to repair the train wreck that is caused when a DNA replication fork runs into a section of DNA damage during the process of DNA replication.

Given the importance of DNA, it is no wonder that the pathways for DNA repair have been largely conserved throughout all three kingdoms of life. That being said, are we all optimized for DNA repair? Could we do it better? This is an important consideration because we live in an environment that can damage DNA in many different ways and, if this damage is not repaired, it can sometimes lead to diseases such as cancer. Now, in *eLife*, Michael Cox of the University of Wisconsin-Madison and co-workers—including Rose Byrne and Audrey Klingele as joint first authors—report that mutations in just three genes can allow the bacteria *Escherichia coli* to improve its ability to repair DNA by a factor of 1000. Might we do the same?

The ability to repair DNA is not the same for all organisms. The bacteria *Deinococcus radiodurans*, for example, is several orders of magnitude more resistant to ionizing radiation than *E. coli*. Why? Does *D. rad* have more or different DNA repair pathways? The *D. rad* genome contains approximately the same repertoire of repair enzymes as *E. coli*. Does this mean that *D. rad* has a higher concentration of repair enzymes? Or perhaps the repair enzymes in *D. rad* are just better at their doing their jobs because they have higher specific activities and/or binding affinities? It is well known that cells respond to DNA damage by expressing DNA repair proteins. Perhaps *D. rad* responds faster than *E. coli*, or perhaps its response has a greater amplitude? Maybe the increased resistance is due to something else, such as the way that DNA is packaged inside the nucleus. Or perhaps *D. rad* is better able to detoxify the reactive oxygen species created by the radiation and/or to cope with damaged proteins?

In 2009, Cox and co-workers posed a different question: is it possible to make *E. coli* more resistant to ionizing radiation? Given the complexity of DNA repair pathways and gene regulation, this was akin to trying to untie a Gordian Knot. To tackle this problem, they used the combined power of in vitro evolution and bacteria genetics and let the bacteria untie the knot for them. They exposed cultures of *E. coli* to devastating amounts of ionizing radiation, isolated those that managed to survive, and repeated this process. After 20 cycles of this process, the cultures of *E. coli* were 3–4 orders of magnitude more resistant to ionizing radiation than the starting *E. coli* strains (Harris et al., 2009).

DNA sequence analysis revealed that each evolved strain had between 40–80 mutations. Moreover, many of these mutations mapped onto genes that were known to be associated with DNA repair, recombination, DNA replication, proteolysis and cell division, although some of the mutations mapped onto genes of unknown function. But which were the important genes and mutations?

Now Cox and co-workers—who are based at the University of Wisconsin, the DOE Joint Genome Institute and Louisiana State University—have answered this question by continuing the *E. coli* evolution experiments and characterizing the DNA sequences of the strains that show high levels of resistance to ionizing radiation (Byrne et al., 2014).

They developed criteria to discern the important mutations from the 40–80 mutations in each strain. This narrowed their focus to single mutations in eight genes: *recA*, *dnaB* and *yfjK* (which are involved in DNA metabolism), *rsxB* and *gsiB* (suppression of oxidative damage), *wcaK* and *nanE* (cell wall biosynthesis), and *glpD* (basic respiration). By transferring these mutations, both singly and in combinations, to fresh strains of *E. coli*, and by removing them from evolved strains of the bacteria, they were able to determine that mutations to three genes*—recA*, *dnaB* and *yfjK*—accounted for about 1000-fold of the increased resistance to ionizing radiation. Moreover, *recA* and *dnaB* were in the same DNA repair pathways, while *yfjK* was in another repair pathway. The other mutations made measureable, but smaller contributions. Cox and co-workers also checked for global changes in the transcriptome, the metablome and metal ion content and found none, so the mutations in these three genes must be the primary reason for the increase in resistance.

The important and overwhelming conclusion from this study is that organisms can increase their resistance to ionizing radiation by 3–4 orders of magnitude and that the increase can be traced back to relatively few mutations. Once the reasons for this increase have been understood in greater detail, these results will have implications for our knowledge of DNA repair in a wide range of species, including humans.
